# Results from a Genome-Wide Association Study (GWAS) in Mastocytosis Reveal New Gene Polymorphisms Associated with WHO Subgroups

**DOI:** 10.3390/ijms21155506

**Published:** 2020-07-31

**Authors:** Bogusław Nedoszytko, Marta Sobalska-Kwapis, Dominik Strapagiel, Magdalena Lange, Aleksandra Górska, Joanne N. G. Oude Elberink, Jasper van Doormaal, Marcin Słomka, Leszek Kalinowski, Marta Gruchała-Niedoszytko, Roman J. Nowicki, Peter Valent, Marek Niedoszytko

**Affiliations:** 1 Department of Dermatology, Venerology and Allergology, Medical University of Gdansk, 80-211 Gdansk, Poland; magdalena.lange@gumed.edu.pl (M.L.); rnowicki@gumed.edu.pl (R.J.N.); 2 Biobank Laboratory, Department of Molecular Biophysics, Faculty of Biology and Environmental Protection, University of Lodz, 90-237 Lodz, Poland; marta.sobalska@biol.uni.lodz.pl (M.S.-K.); marcin.slomka@biol.uni.lodz.pl (M.S.); 3 Biobanking & Molecular Biotechnologies Resources Infrastructures Poland (BBMRI.pl) Consortium, 50-367 Wrocław, Poland; lekal@gumed.edu.pl; 4 Department of Allergology, Medical University of Gdansk, 80-211 Gdansk, Poland; agorska@gumed.edu.pl (A.G.); mnied@gumed.edu.pl (M.N.); 5 Department of Allergology, Internal Medicine, University Medical Center Groningen, University of Groningen, 9713 GZ Groningen, The Netherlands; j.n.g.oude.elberink@umcg.nl (J.N.G.O.E.); j.j.van.doormaal@int.umcg.nl (J.v.D.); 6 Groningen Research Institute for Asthma and COPD, University of Groningen, University Medical Center Groningen, 9713 GZ Groningen, The Netherlands; 7 Department of Medical Laboratory Diagnostics, Medical University of Gdansk, 80-211 Gdansk, Poland; 8 Department of Mechanics of Materials and Structures, Gdansk University of Technology, 80-223 Gdansk, Poland; 9 Department of Clinical Nutrition, Medical University of Gdansk, 80-211 Gdansk, Poland; mg@gumed.edu.pl; 10 Division of Hematology and Hemostaseology, Department of Internal Medicine I, Medical University of Vienna, 18-20 Vienna, Austria; peter.valent@meduniwien.ac.at; 11 Ludwig Boltzmann Institute for Hematology & Oncology, Medical University of Vienna, Medical University of Vienna, Waehringer Guertel, 18-20 Vienna, Austria

**Keywords:** KIT, tryptase, prognostication, gene polymorphisms

## Abstract

Mastocytosis is rare disease in which genetic predisposition is not fully understood. The aim of this study was to analyze associations between mastocytosis and single nucleotide polymorphisms (SNPs) by a genome-wide association study (GWAS) approach. A total of 234 patients were enrolled in our study, including 141 with cutaneous mastocytosis (CM; 78 children and 63 adults) and 93 with systemic mastocytosis (SM, all adults). The control group consisted of 5606 healthy individuals. DNA samples from saliva or blood were genotyped for 551 945 variants using DNA microarrays. The prevalence of certain SNPs was found to vary substantially when comparing patients and healthy controls: rs10838094 of *5OR51Q1* was less frequently detected in CM and SM patients (OR = 0.2071, *p* = 2.21 × 10^−29^), rs80138802 in *ABCA2* (OR = 5.739, *p* = 1.98 × 10^−28^)*,* and rs11845537 in *OTX2-AS1* (rs11845537, OR = 6.587, *p* = 6.16 × 10^−17^) were more frequently detected in CM in children and adults. Additionally, we found that rs2279343 in *CYP2B6* and rs7601511 in *RPTN* are less prevalent in CM compared to controls. We identified a number of hitherto unknown associations between certain SNPs and CM and/or SM. Whether these associations are clinically relevant concerning diagnosis, prognosis, or prevention remains to be determined in future studies.

## 1. Introduction

Mastocytosis is a heterogeneous group of diseases defined by the abnormal accumulation of clonal mast cells (MC) in the skin, bone marrow, and/or other visceral organs. The diagnosis of systemic mastocytosis (SM) is based on WHO criteria, including the basal serum tryptase level, histopathological and immunophenotypic (CD2/CD25) features of MCs, and somatic *KIT* mutations in codon 816. Mastocytosis can be divided into 7 variants: cutaneous mastocytosis (CM), indolent systemic mastocytosis (ISM), smoldering SM (SSM), SM with an associated hematological neoplasm (SM-AHN), aggressive SM (ASM), MC leukemia (MCL), and MC sarcoma (MCS) [1].

The majority of children with CM have a favorable prognosis. In contrast, in adults, mastocytosis usually presents as SM, and sometimes progresses to an aggressive disease [2,3,4,5,6,7,8].

In most patients with SM, somatic mutations in the *KIT* gene are found, the most prevalent in SM being D816V (rs121913507, D [GAC] > V [GTC]). The protein product of *KIT* (KIT = CD117) is a transmembrane receptor for the stem cell factor, a major regulator of MC differentiation and survival. The *KIT* D816V mutation results in SCF-independent differentiation of MCs. KIT D816V is detected in more than 80% of all adults with SM. In children with CM, this mutation is less frequent (35%) and other mutations, located in gene regions encoding the external cellular domain of KIT, are more commonly found in childhood patients (45%) [7,9,10].

Genetic studies performed so far in CM and SM focused on the gene polymorphisms of cytokines and their receptors (*IL-13, IL-6, IL6R, IL-31, IL4R, VEGFA*), *TLRs* and variants of the *KIT* gene. These studies have shown that some cytokine or cytokine receptor gene polymorphisms may be associated with the presence of SM and/or CM [11,12,13,14,15,16]. Other studies performed in ISM found associations of SM disease with SNPs in *RAB27A, ETS1, ITGB1, MLL3,* and *ITGAV* genes [17].

In advanced SM, additional recurrent somatic mutations involving genes encoding factors regulating splicing, signaling transmission, and epigenetic processes have recently been described [17,18,19,20,21]. The most frequently mutated genes are *TET-2*, *DNMT3A*, and *ASXL1* [18,19,20,21,22].

In our study, we analyzed the genetic background of CM and SM using a genome wide association technique.

## 2. Results

### 2.1. Comparison of All Mastocytosis Patients with the Controls

**Figure 1:**
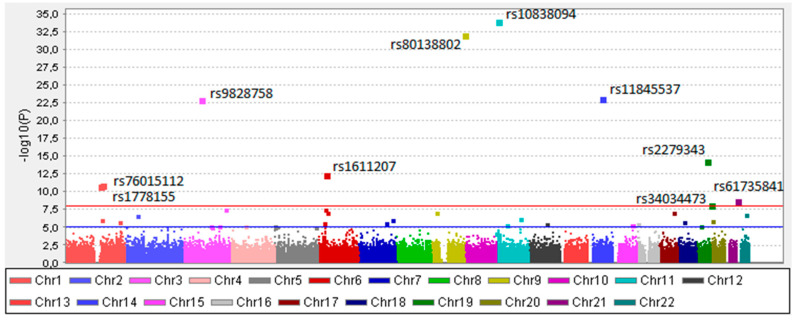
The “Manhattan” plot for the genome-wide association study of all analyzed mastocytosis patients. On the x-axis, each color represents different chromosomes. The log_10_ of the unadjusted *p*-values (without multiple testing corrections) are shown at the y-axis. The blue line indicates the suggestive association threshold (10^−5^), while red line indicates GWAS significant threshold (10^−8^).

Of the 281 811 analyzed SNPs, 9 showed a statistically different frequency in mastocytosis patients. Four of the SNPs were more frequently found in mastocytosis patients compared to controls: rs80138802 in *ABCA2 (OR =* 5.739, (95% CI; 4.156–7.925), *p* = 1.98 × 10^−27^), rs11845537 in *OTX2-AS1* (OR = 5.625, (95% CI; 3.859–8.199), *p* = 1.60 × 10^−18^), rs1611207 in *HLA-V* (OR *=* 2.105, (95% CI; 1.717–2.581), *p* = 7.25 × 10^−8^), and rs1778155 in *PDE4DIP (OR =* 2.032, (95% CI; 1.649–2.504), *p* = 3.26 × 10^−6^) genes (Table 1, Figure 1).

Five polymorphisms were found to be less frequently detectable in mastocytosis than in controls: rs61735841 in *FTCD* (OR = 0.026; 95% CI 0.003612–0.1833; *p* = 4.34 × 10^−5^), rs10838094 in *OR51Q1* (OR = 0.2071; 95% CI 0.1572–0.2728; *p* = 2.21 × 10^−29^), rs2279343 in *CYP2B6*, OR = 0.2795; 95%CI; 0.199–0.3924; *p* = 2.32 × 10^−10^), rs76015112 in *RPTN* (OR = 2.965; 95% CI 0.205–0.4289; *p* = 2.94 × 10^−7^) genes and rs9828758 (OR = 0.1467; 95% CI 0.095–0.23; *p* = 2.94 × 10^−7^) near RP11 gene (Table 1, Figure 1, Figures S3 and S4).

Tables S1–S5 provides an analysis of the frequency of genotypes, alleles, and their mode of inheritance and expression frequencies in SM and CM patients. Apart from the rs80138802 A > C, *ABCA2* gene polymorphism, where a recessive mode of inheritance was found, the other gene polymorphisms showed a dominant mode of inheritance.

### 2.2. Comparison of Patients with SM and Controls

When analyzing patients with SM (88 ISM and 3 SSM), only 3 SNPs were identified to be more or less prevalent in SM patients compared to the controls. Two SNPs, rs2857596 (OR = 2.582 (95% CI; 1.909–3.492), *p* = 2.45 ×10^−5^) located near *NRC3* and rs498404 (OR = 2.697 (95% CI; 0.1634–3.716) *p* = 2.46 × 10^−5^) near *TTC398* genes were more frequently detected, whereas rs10838094 in *OR51Q1* (OR = 0.2761 (95% CI; 0.1864–0.4088, *p* = 1.83 × 10^−6^) was less frequently detected in SM patients compared to controls (Table 2, Figures S1 and S2).

### 2.3. Comparison of CM Patients with Controls

We found that 6 SNPs were differently expressed in CM (both children and adults) patients compared to the controls. Two SNPs were more frequently detected: *ABCA2* (rs80138802, OR = 6.969 (95% CI; 4.749–10.23), *p* = 4.20 × 10^−25^) and *OTX2-AS1* (rs11845537, OR = 6.587 (95% CI; 4.235–10.24) *p* = 6.16 × 10^−17^), and 4 SNPs were less frequently identified compared to controls: *OR51B5* (rs10838094, OR = 0.1646 (95% CI; 0.1119–0.2421), *p* = 3.28 × 10^−20^)*, CYP2B6* (rs2279343, OR = 0.181 (95% CI; 0.1073–0.3053), *p* = 3.26 × 10^−8^)*, RPTN* (rs76015112, OR = 0.1427 (95% CI; 0.0732–0.2783), *p* = 1.35 × 10^−6^)^,^ and rs9828758 (OR = 0.1454 (95% CI; 0.0862–0.2452), *p* = 3.68 × 10^−12^) located near the *RP11* gene (Table 3).

A comparison of the results found in CM or SM patients with controls indicated that only rs10838094 in *OR51B5* gene is less frequent in both groups, with strong statistic p value (2.40 × 10^−25^ for CM and 6.71 × 10^−12^ for SM) (Table 2 and Table 3, Figures S1 and S2). 

The SNPs rs2857596 and rs498404 were more prevalent in SM, whereas rs80138802 in *ABCA2* and rs11845537 in *OTX2-AS1* genes were more frequently detected in CM patients. Additionally, we found that in CM patients (but not SM patients), the polymorphisms rs2279343 in *CYP2B6* and rs76015112 in *RPTN* genes were statistically less frequently detected than in controls.

### 2.4. Comparison of Children and Adult Patients with Cutaneous Mastocytosis with Controls

When we separately compared adults with CM (adCM) and children with CM (chCM) with the control group, we found differences in the frequency of several SNPs. In both groups, two polymorphisms were expressed more frequently than in the control group (rs80138802 in the *ABCA2* gene and rs11845537 in the *OTX2-AS1* gene). Additionally, in children with CM, 2 SNPs were statistically less frequent (rs10838094 in *OR51Q1* gene and rs9828758) (Table 4A,B).

We also compared our data with previously published results. In particular, we examined the genes of cytokines, growth factors, and transcription factors described as potential factors associated with mastocytosis (Table S6), genes involved in epigenetic processes, transcription and cell proliferation (Table S7), and genes where the differences in expression were described in indolent systemic mastocytosis (Table S11). Most of the analyzed genes showed significant differences in prevalence when analyzed separately. However, after correcting the data for false discovery rates and multivariate testing, no significant correlations were found (Tables S8–S10 and S12–S14).

## 3. Discussion 

In recent years, genetic background has been implicated in the pathogenesis of CM and SM. However, only little is known about specific genes and SNPs contributing to the clinical course and prognosis in mastocytosis patients. The results of our study suggest that there is an association between mastocytosis and 9 SNPs which were not described in mastocytosis contexts so far. Four SNPs were more prevalent (in *ABCA2*, *OTX2-AS1, HLA-V,* and *PDE4DIP* genes) and 5 were less prevalent (in *RPTN, CYP2B6, OR51Q1, FTCD,* and rs9828758 near *RP11* genes) in mastocytosis patients compared to a control cohort. Further studies on these SNPs may lead to new insights into the pathogenesis of mastocytosis and the development of new preventive or interventional medication.

Genotypes CC and CA of the *ABCA2* (rs80138802 A > C) gene were more frequent in patients than in the controls and were inherited in recessive mode. These polymorphisms were especially more frequent in CM patients. An attractive hypothesis would be that patients with the *ABCA2* (rs80138802 A > C) genotype have an increased risk of developing CM. An association between this genotype and adulthood CM, as well as childhood CM, was found. In the SM group of patients, this allele is also more prevalent, but the association did not reach statistical significance (*p* = 0.001482, Table 2).

*ABCA1* and *ABCA2* genes *(ABCA*—ATP Binding Cassette Subfamily A Member) encode the membrane transporters involved in lipid metabolism and drug elimination. The overexpression of ATP-binding cassette transporters is a major adaptive advantage used by tumor cells to evade the accumulation of cytotoxic agents. The expression of ABC transporters has thus been linked with multidrug resistance phenotypes through the efflux of small drugs via ATP-dependent transport [23,24]. One of the drugs affected is imatinib, which is sometimes applied in patients with KIT D816V-negative SM [2,25,26,27].

However, the role of ABC transporters has not been studied in detail in the mastocytosis context so far. In particular, it remains unknown whether this gene polymorphism plays a role in the resistance of neoplastic (mast) cells against TKI D816V-targeting drugs, like midostaurin or avapritinib.

Several other gene SNPs were also found to be more prevalent in patients with CM and/or SM, including OTX2-AS1 (rs11845537 G > A), PDE4DIP (rs1778155 C > T), HLA-V (29759876 A > G), RPTN (152129094 G > A), CYP2B6 (rs2279343 G > A, OR51Q1 (rs10838094 A > G), and FTCD (47558552 A > G). So far, little is known about the function and clinical implications of these genes, and no studies exploring the impact of these SNPs in the mastocytosis context are available.

Some of the associations were striking. For example, polymorphism rs11845537 G > A of the *OTX2-AS1* gene was frequently observed both in adults and in children with CM. In fact, patients with an A allele were found to have a 6 to 7 times higher prevalence of mastocytosis compared to the controls. The genotype AA and AG were more frequent in both children and adult patients and the allele A was also found to be inherited in a dominant mode.

OTX2 antisense RNA 1 (head to head) long-noncoding RNA, is a transcription factor known to play an important role in controlling the expression of the *OTX2-AS1* gene. OTX2 is an epigenetic factor, playing a role in transcription repression, chromatin remodeling, histone modification, and cell cycle regulation [24,28]. Increased amounts of OTX2-AS1 were observed in exosomes in bladder cancers [28]. Somatic mutation of the genes which play a role in epigenetic regulation of gene expression are described in the advanced form of mastocytosis. So far, however, the potential role and impact of the *OTX2-AS1* gene in mastocytosis remain unknown [29].

So far, several gene SNPs located in cytokine genes, their receptors, and toll-like receptors have been identified in mastocytosis [11,12,13,14,15]. Other genes were described in the context of epigenetic processes regulation in mastocytosis [19,20,21,22]. We performed analyses of those genes in our study. Most of the analyzed genes showed significant differences in the prevalence when analyzed separately. However, the analysis using the correction for the false discovery rate showed insignificant results.

Our data add another set of potentially important and clinically interesting gene SNPs in mastocytosis. However, it is difficult to define the real impact of these SNPs for several reasons. First, the number of patients analyzed in each subgroup was too small to draw definitive conclusions regarding the pathogenetic impact of these SNPs. Second, our data need to be verified and confirmed in other independent prospective studies. Third, preclinical models exploring the potential functional role of these SNPs are lacking. Therefore, we regard our study as an interesting starting point of biomedical research where each of the identified gene SNP that clusters in distinct forms of CM and/or SM should be validated in future studies.

In conclusion, the results of our study suggest associations between mastocytosis variants and SNP which have not been described so far and which may potentially have clinical and prognostic implications. Our results are promising but require further validation in larger studies with several more patients with mastocytosis from other EU countries. Additional future research using gene sequencing techniques and in vitro studies on the functional role of identified SNPs on mast cell physiology are needed and planned. 

## 4. Patients and Methods

### 4.1. Patient Groups 

The patients included in this study were recruited between 2008 and 2019 from patients seen at the Medical University of Gdansk in Poland, the University Medical Center in Groningen in the Netherlands, and the Medical University in Vienna, Austria. 

A total of 234 patients were included. Of these patients, 141 were diagnosed with CM and 93 with SM based on WHO criteria [1,2,3,4]. Of the patients with CM (all from Poland), 78 were children (42 boys and 36 girls, mean age 4.2 ± 3.5 years) and 63 were adults (50 females and 13 males, age range 18–72 years; mean age 37.6 ± 13.7 years), of which 74 (94.9%) presented with maculopapular cutaneous mastocytosis (MPCNM) and 4 (5.1%) with diffuse cutaneous mastocytosis (DCM). All adult patients with CM presented with MPCM; adult patients were checked for SM and they did not fulfil SM criteria. Of the 93 patients with SM (29 males and 64 females, age range 25–76 years; mean age 40.2 ± 8.2), 88 were diagnosed with ISM, 3 with smoldering systemic mastocytosis (SSM), one with ASM, and one with MCL. 

Of the 93 SM patients, 20 (all with ISM) were from the University Medical Center in Groningen in the Netherlands, 23 were from the Medical University in Austria (MCL, ASM, SSM), and 50 patients (all with ISM) were from Poland. 

The study protocol was approved by the Independent Bioethics Committee for Scientific Research of the Medical University of Gdansk, (NKBBN/331/2017), Groningen (METc 2008/340), and Vienna (1184/2014). All subjects provided written, informed consent prior to their participation in the study.

### 4.2. Control Group

Control donors were recruited between 2010 and 2012 within the research project TESTOPLEK and registered as the POPOLOUS collection at the Biobank Lab of The Department of Molecular Biophysics of The University of Lodz, Poland. Each donor gave written, informed consent to participate. Saliva was collected into Oragene OG-500 DNA collection/storage receptacles (DNA Genotek, Kanata, Canada) from each individual. The approval for this study was obtained from The University of Lodz’s Review Board (32/KBBN-UL/I/2018). All procedures were performed in accordance with the Declaration of Helsinki (ethical principles for medical research involving human subjects) [30,31].

From over 6047 adult individuals throughout Poland, a total of 5606 participants were involved in a matched control group of the study. The exclusion criteria were diabetes, myeloid disorders, bone marrow transplantation, and cancer. There were 2860 (51%) females and 2746 (49%) males, aged from 22 to 77 years (42.86 ± 14.85 and 42.03 ± 14.72, respectively). 

Based on mitochondrial DNA (mtDNA) studies Poles can be considered as genetically homogenous, with the same pattern as other population within European countries (e.g., The Netherlands, Austria, Spain, Portugal, Sardinia and Russia) [32].

### 4.3. DNA Isolation

The samples of the peripheral blood (patient’s) or saliva (control) were collected and stored at −80 °C.

Genomic DNA was extracted from 200 µL of blood using the Magna Pure LC 2.0 (Roche, Rotkreuz, Switzerland) with the DNA Isolation Kit I High Performance protocol launched.

Genomic DNA from saliva samples was manually isolated from 500 µL using the manufacturer’s instructions (PrepitL2P, PD-PR-052, DNA Genotek, Kanata, ON, Canada). The elution volume was 50 µL.

A total of 234 DNA samples from patients and 5606 from control group were quantified using broad range Quant-iT™ dsDNA Broad Range Assay Kit (Invitrogen™, Carlsbad, CA, USA). All DNA samples underwent quality control in PCR reaction for sex determination [33].

### 4.4. Microarrays Analysis

DNA samples were genotyped for 558 231 SNPs using the 24 × 1 Infinium HTS Human Core Exome (Illumina Inc., San Diego, CA, USA) microarrays according to the protocol provided by the manufacturer.

### 4.5. Statistical Methods

A preliminary statistical analysis of the database containing the results of the genotyping of 5840 individuals for 558,231 SNPs was carried out in the PLINK 1.9 program. From the statistical analysis, 276,420 SNPs were excluded because:

(1) 16,119 variants were removed due to missing genotype data;
(2) 3340 variants were removed due to Hardy–Weinberg exact test;
(3) 256,961 variants were removed due to minor allele threshold.

To discover and validate genetic risk-factors for mastocytosis, the chi-square statistic with odds ratio and 95% confidence interval for 2 × 2 contingency tables were calculated using PLINK 1.9. To adjust for multiple testing, the thresholds of *p* < 1 × 10^−5^ and *p* < 5 × 10^−8^ were used for suggestive and genome-wide significant associations, respectively [34]. Regional associations were visualized with the Haploview or LocusZoom web tool (http://locuszoom.org/).

## 5. Conclusions

The results of our study suggest an association between mastocytosis and 9 SNPs which were not described in mastocytosis so far, which may be important for the pathogenesis of the disease. Moreover, 4 SNPs were more prevalent (*ABCA2*, *OTX2-AS1, HLA-V,* and *PDE4DIP)* and 5 were found to have less prevalence (*RPTN, CYP2B6, OR51Q1, FTCD* genes, and rs9828758).

In the future, for each identified variant, we plan to perform in vitro evaluation of its effects on mast cell physiology, and, in case if these variant would be found non-causal, to sequence adjacent loci for other functional variants linked to the reported ones

## Figures and Tables

**Table 1:** Comparison of the frequency of single nucleotide polymorphisms (SNPs) in mastocytosis patients (*n* = 234) and control groups (*n* = 5606).

	CHR	SNP	BP	A1	F_A	F_U	A2	CHISQ	P	OR	SE	L95	U95	BONF	FDR_BH	Gene	Description
1	11	rs10838094	5443893	A	0.1261	0.4106	G	151.5	8.10 × 10^−35^	0.2071	0.1406	0.1572	0.2728	2.21 × 10^−29^	2.21 × 10^−29^	*OR51Q1*	olfactory receptor family 51 subfamily B member OR51Q1
2	9	rs80138802	139915940	C	0.125	0.02429	A	142.6	7.22 × 10^−33^	5.739	0.1646	4.156	7.925	1.98 × 10^−27^	9.88 × 10^−28^	*ABCA2*	protein_coding
3	14	rs11845537	57446273	A	0.07725	0.01467	G	101.9	5.85 × 10^−24^	5.625	0.1923	3.859	8.199	1.60 × 10^−18^	5.33 × 10^−19^	*OTX2-AS1*	OTX2 antisense RNA 1. transcription factor controlling expression of OTX2 gene.
4	3	rs9828758	73718136	T	0.05729	0.293	C	101.1	8.65 × 10^−24^	0.1467	0.2206	0.09518	0.226	2.36 × 10^−18^	5.91 × 10^−19^	*Near RP11*	Promoter
5	19	rs2279343	41515263	G	0.08447	0.2482	A	61.59	4.24 × 10^−15^	0.2795	0.1732	0.199	0.3924	1.16 × 10^−9^	2.32 × 10^−10^	*CYP2B6*	protein_coding
6	6	rs1611207	29759876	A	0.5641	0.3807	G	53.45	2.65 × 10^−13^	2.105	0.104	1.717	2.581	7.25 × 10^−8^	1.21 × 10^−8^	*HLA-V*	pseudogene
7	1	rs76015112	152129094	G	0.07488	0.2144	A	46.88	7.53 × 10^−12^	0.2965	0.1883	0.205	0.4289	2.06 × 10^−6^	2.94 × 10^−7^	*RPTN*	protein_coding
8	1	rs1778155	144874815	T	0.6282	0.454	C	45.99	1.19 × 10^−11^	2.032	0.1066	1.649	2.504	3.26 × 10^−6^	4.07 × 10^−7^	*PDE4DIP*	protein_coding
9	21	rs61735841	47558552	A	0.00236	0.08414	G	36.63	1.43 × 10^−9^	0.02573	1.002	0.003612	0.1833	0.0003907	4.34 × 10^−5^	*FTCD*	protein_coding
10	19	rs34034473	58132106	C	0.1116	0.05008	A	33.8	6.10 × 10^−9^	2.383	0.1538	1.762	3.221	0.001669	0.0001669	*ZNF134*	protein_coding
11	3	rs496250	180627303	T	0.2271	0.1323	C	30.59	3.19 × 10^−8^	1.927	0.1206	1.521	2.441	0.008734	0.0007551	*FXR1*	protein_coding
12	6	rs3131382	31707730	A	0.07906	0.03194	G	30.51	3.31 × 10^−8^	2.602	0.1795	1.83	3.699	0.009062	0.0007551	*MSH5*	protein_coding
13	9	rs4316218	24906802	C	0.4145	0.2978	T	29.03	7.13 × 10^−8^	1.67	0.09608	1.383	2.016	0.01949	0.001499		
14	17	rs8081489	60285205	C	0.04915	0.01604	A	28.65	8.69 × 10^−8^	3.171	0.2275	2.03	4.952	0.02377	0.001593		
15	6	rs45487695	39895263	T	0.03419	0.00901	C	28.64	8.74 × 10^−8^	3.893	0.2737	2.277	6.657	0.02389	0.001593	*MOCS1*	Molybdenum Cofactor Synthesis 1
16	22	rs138366	38789936	A	0.3739	0.5004	G	27.33	1.72 × 10^−7^	0.5963	0.09989	0.4902	0.7252	0.04689	0.002931	*CSNK1E*	protein_coding
17	2	rs13015643	52369737	C	0.1416	0.07551	A	27.15	1.88 × 10^−7^	2.02	0.1376	1.543	2.645	0.05143	0.003025	*AC007682.1*	lincRNA
18	11	rs7931273	100848015	T	0.2137	0.3236	C	24.96	5.85 × 10^−7^	0.5681	0.1146	0.4539	0.7112	0.1601	0.008893	*ARHGAP42*	protein_coding
19	1	rs3014818	153393554	C	0.09402	0.04476	T	24.28	8.34 × 10^−7^	2.215	0.1655	1.601	3.063	0.2281	0.012	*S100A7A*	protein_coding
20	7	rs4987668	142572649	C	0.0636	0.02548	T	24.03	9.51 × 10^−7^	2.598	0.202	1.748	3.86	0.26	0.013	*TRPV6*	protein_coding

Table legend: CHR—chromosome, BP–base pair based on the human reference genome GRCh37, A1—minor allele, A2—major allele, F_A—frequency of A1 allele in patients, F_U—frequency of A1 allele in controls, CHISQ—basic allelic test chi-square (1df), P—*p*-value for CHISQ, OR—odds ratio, SE—standard error, L95 and U95—95% confidence interval for odds ratio, lower bound and upper bound, respectively, BONF—Bonferroni single-step adjusted *p*-values, FDR_BH—Benjamini & Hochberg (1995) step-up false discovery rate control.

**Table 2:** Comparison of the frequency of SNPs in patients with systemic disease (*n* = 93) and control groups (*n* = 5606).

	CHR	SNP	BP	A1	F_A	F_U	A2	CHISQ	P	OR	SE	L95	U95	BONF	FDR_BH	Gene
1	11	rs10838094	5443893	A	0.1613	0.4106	G	47.11	6.71 × 10^−12^	0.2761	0.2003	0.1864	0.4088	1.83 × 10^−6^	1.83 × 10^−6^	*OR51Q1*
2	6	rs2857596	31567422	C	0.4483	0.2394	A	40.69	1.79 × 10^−10^	2.582	0.1541	1.909	3.492	4.89 × 10^−5^	2.45 × 10^−5^	*NRC3*
3	9	rs498404	15347022	G	0.2989	0.1365	T	39.88	2.70 × 10^−10^	2.697	0.1634	0.1634	3.716	7.38 × 10^−5^	2.46 × 10^−5^	*TTC398*
4	12	rs1479010	78408770	T	0.629	0.4216	C	32.23	1.37 × 10^−8^	2.327	0.153	1.724	3.14	0.003748	0.0009369	*NAV3*
5	3	rs9828758	73718136	T	0.05833	0.293	C	31.71	1.79 × 10^−8^	0.1495	0.3901	0.0696	0.3211	0.004893	0.0009786	Near *RP11*
6	16	rs9937881	1297071	T	0.3763	0.2089	C	30.77	2.91 × 10^−8^	2.286	0.1531	1.693	3.086	0.007952	0.001325	intergenic variant nearest *tryptase-α and δ* genes
7	9	rs80138802	139915940	C	0.09259	0.02429	A	30.25	3.79 × 10^−8^	4.099	0.2779	2.378	7.068	0.01037	0.001482	*ABCA2*
8	6	rs45487695	39895263	T	0.04839	0.00901	C	29.63	5.23 × 10^−8^	5.593	0.3563	2.782	11.24	0.0143	0.001647	*MOCS1*
9	4	rs1820510	159873196	A	0.09677	0.02863	G	29.44	5.76 × 10^−8^	3.635	0.2544	2.208	5.985	0.01575	0.001647	*C4orf45*
10	8	rs954009	33195455	C	0.4086	0.2375	T	29.36	6.02 × 10^−8^	2.219	0.1508	1.651	2.982	0.01647	0.001647	*FUC10*
11	6	rs17404123	65303100	C	0.2308	0.1065	T	28.58	8.98 × 10^−8^	2.516	0.1786	1.773	3.571	0.02455	0.002231	*EYS*
12	6	exm529784	31084964	T	0.4194	0.249	C	28.18	1.11 × 10^−7^	2.178	0.1502	1.623	2.924	0.03027	0.002523	*CDSN*
13	9	rs2230808	107562804	A	0.4176	0.2469	G	27.85	1.31 × 10^−7^	2.187	0.1519	1.624	2.945	0.03584	0.002757	*ABCA1*
14	8	rs12546032	33187102	A	0.4032	0.2371	G	27.69	1.42 × 10^−7^	2.174	0.1511	1.617	2.923	0.03885	0.002775	*FUC10*
15	4	rs2276938	159780244	G	0.08696	0.02595	C	25.7	3.99 × 10^−7^	3.574	0.2683	2.113	6.047	0.1091	0.006891	*FNIP2*
16	6	rs807816	21264120	G	0.1989	0.09049	A	25.68	4.03 × 10^−7^	2.496	0.1866	1.731	3.598	0.1103	0.006891	
17	1	rs12402123	181490018	T	0.1398	0.05467	C	25.05	5.60 × 10^−7^	2.81	0.2155	1.842	4.286	0.1531	0.009007	CACNA1E
18	6	rs16895517	65300143	C	0.2174	0.1037	G	24.77	6.46 × 10^−7^	2.4	0.1814	1.682	3.425	0.1766	0.009812	*EYS*
19	1	rs35701577	3522561	C	0.3132	0.1731	T	24.32	8.18 × 10^−7^	2.179	0.1618	1.587	2.991	0.2236	0.01177	*MEGF6*
20	6	rs3131382	31707730	A	0.09677	0.03194	G	24.1	9.15 × 10^−7^	3.247	0.2538	1.975	5.34	0.2501	0.0125	*MSH5*

Table legend: CHR—chromosome, BP—base pair based on the human reference genome GRCh37, A1—minor allele, A2—major allele, F_A—frequency of A1 allele in patients, F_U—frequency of A1 allele in controls, CHISQ—basic allelic test chi-square (1df), P—*p*-value for CHISQ, OR—odds ratio, SE—standard error, L95 and U95—95% confidence interval for odds ratio, lower bound and upper bound, respectively, BONF—Bonferroni single-step adjusted *p*-values, FDR_BH—Benjamini & Hochberg (1995) step-up false discovery ra3.te control.

**Table 3:** Comparison of the frequency of SNPs in patients with cutaneous mastocytosis (*n* = 141) and control groups (*n* = 5606).

	CHR	SNP	BP	A1	F_A	F_U	A2	CHISQ	P	OR	SE	L95	U95	BONF	FDR_BH	Gene
1	9	rs80138802	139915940	C	0.1478	0.02429	A	132	1.53 × 10^−30^	6.969	0.1957	4.749	10.23	4.20 × 10^−25^	4.20 × 10^−25^	*ABCA2*
2	11	rs10838094	5443893	A	0.1028	0.4106	G	108.2	2.40 × 10^−25^	0.1646	0.197	0.1119	0.2421	6.55 × 10^−20^	3.28 × 10^−20^	*OR51Q1*
3	14	rs11845537	57446273	A	0.08929	0.01467	G	92.49	6.75 × 10^−22^	6.587	0.2253	4.235	10.24	1.85 × 10^−16^	6.16 × 10^−17^	*OTX2-AS1*
4	3	rs9828758	73718136	T	0.05682	0.293	C	70.19	5.38 × 10^−17^	0.1454	0.2667	0.0862	0.2452	1.47 × 10^−11^	3.68 × 10^−12^	*Near RP11*
5	19	rs2279343	41515263	G	0.05639	0.2482	A	51.86	5.97 × 10^−13^	0.181	0.2667	0.1073	0.3053	1.63 × 10^−7^	3.26 × 10^−8^	*CYP2B6*
6	1	rs7601511	152129094	G	0.0375	0.2144	A	44.2	2.97 × 10^−11^	0.1427	0.3406	0.07322	0.2783	8.13 × 10^−6^	1.35 × 10^−6^	*RPTN*
7	6	rs1611207	29759876	A	0.5526	0.3807	G	32.46	1.22 × 10^−8^	2.01	10.24	1.573	2.567	0.003335	0.0004764	*HLA-V*
8	1	rs1778155	144874815	T	0.6203	0.454	C	28.93	7.52 × 10^−8^	1.965	0.1278	1.53	2.524	0.02055	0.002569	*PDE4DIP*
9	6	rs13195402	26463575	T	0.09574	0.03641	G	26.47	2.67 × 10^−7^	2.802	0.209	1.86	4.221	0.07301	0.008113	*BTN2A1*
10	1	rs973253	231520874	C	0.4362	0.2992	T	24.44	7.65 × 10^−7^	1.812	0.1218	1.427	2.3	0.2093	0.02093	*EGLN1*

Table legend: CHR—chromosome, BP–base pair based on the human reference genome GRCh37, A1—minor allele, A2—major allele, F_A—frequency of A1 allele in patients, F_U—frequency of A1 allele in controls, CHISQ—Basic allelic test chi-square (1df), P—*p*-value for CHISQ, OR—odds ratio, SE—Standard Error, L95 and U95—95% confidence interval for odds ratio, lower bound and upper bound, respectively, BONF—Bonferroni single-step adjusted *p*-values, FDR_BH—Benjamini & Hochberg (1995) step-up False Discovery Rate control.

**Table 4:** Comparison of the frequency of SNPs in the adult (**A**) and child patients (**B**) with cutaneous mastocytosis (*n* = 63) and the control groups (*n* = 5606).

	CHR	SNP	BP	A1	F_A	F_U	A2	CHISQ	P	OR	SE	L95	U95	BONF	FDR_BH	Overlapped Gene
**A. Adults with CM (*n* = 65)**																
1	9	rs80138802	139915940	C	0.1471	0.02429	A	61.54	4.33 × 10^−15^	6.926	0.2863	3.952	12.14	1.18 × 10^−9^	1.18 × 10^−9^	ABCA2
2	14	rs11845537	57446273	A	0.09524	0.01467	G	52.42	4.49 × 10^−13^	7.073	0.3146	3.818	13.1	1.23 × 10^−7^	6.14 × 10^−8^	OTX2-AS1
3	11	rs10838094	5443893	A	0.1429	0.4106	G	36.98	1.20 × 10^−9^	0.2393	0.2553	0.1451	0.3947	0.0003271	0.000109	OR51Q1
4	7	rs41271217	158707017	G	0.09524	0.02292	A	28.13	1.13 × 10^−7^	4.487	0.31	2.444	8.238	0.03098	0.007746	WDR60
**B. Children with CM (*n* = 78)**																
1	9	rs80138802	139915940	C	0.1484	0.02429	A	77.91	1.08 × 10^−18^	7.003	0.2561	4.239	11.57	2.95 × 10^−13^	2.95 × 10^−13^	ABCA2
2	11	rs10838094	5443893	A	0.07051	0.4106	G	73.76	8.81 × 10^−18^	0.1089	0.3133	0.05893	0.2013	2.41 × 10^−12^	1.20 × 10^−12^	OR51Q1
3	3	rs9828758	73718136	T	0.01923	0.293	C	56.08	6.97 × 10^−14^	0.04732	0.5834	0.01508	0.1485	1.91 × 10^−8^	6.36 × 10^−9^	Near RP11
4	14	rs11845537	57446273	A	0.08442	0.01467	G	47.71	4.93 × 10^−12^	6.195	0.3015	3.431	11.18	1.35 × 10^−6^	3.37 × 10^−7^	OTX2-AS1
5	1	rs76015112	152129094	G	0	0.2144	A	34.82	3.61 × 10^−9^	0	inf	0	nan	0.0009877	0.0001975	RPTN
6	3	rs3218642	121207637	T	0.1026	0.02792	G	30.5	3.34 × 10^−8^	3.979	0.2701	2.344	6.755	0.00914	0.001472	POLQ
7	18	rs8088340	5836382	C	0.07692	0.01748	T	30.27	3.77 × 10^−8^	4.684	0.309	2.556	8.582	0.0103	0.001472	RP11-945C19.1
8	8	rs1909936	82245451	C	0.1026	0.02855	T	29.38	5.94 × 10^−8^	3.889	0.2699	2.291	6.601	0.01624	0.00203	
9	19	rs2279343	41515263	G	0.05479	0.2482	A	29.09	6.92 × 10^−8^	0.1756	0.3643	0.08598	0.3586	0.01892	0.002102	CYP2B6
10	3	rs2728007	1587151	C	0.09615	0.02755	T	26.15	3.16 × 10^−7^	3.755	0.2777	2.179	6.47	0.08645	0.008645	
11	13	rs9530313	74998658	A	0.141	0.05122	G	24.97	5.83 × 10^−7^	3.041	0.234	1.923	4.811	0.1593	0.01448	LINC00381

Table legend: CHR—chromosome, BP—base pair based on the human reference genome GRCh37, A1—minor allele, A2—major allele, F_A—frequency of A1 allele in patients, F_U—frequency of A1 allele in controls, CHISQ—basic allelic test chi-square (1df), P—*p*-value for CHISQ, OR—odds ratio, SE—standard error, L95 and U95—95% confidence interval for odds ratio, lower bound and upper bound, respectively, BONF—Bonferroni single-step adjusted *p*-values, FDR_BH—Benjamini & Hochberg (1995) step-up false discovery rate control.

## Supplementary Materials

The following are available online at https://www.mdpi.com/1422-0067/21/15/5506/s1.
