# Supplementary material for: Results from a Genome-Wide Association Study (GWAS) in Mastocytosis Reveal New Gene Polymorphisms Associated with WHO Subgroups
Source: Int J Mol Sci. 2020 Jul 31;21(15):5506. doi: 10.3390/ijms21155506 (PMC7432708; doi:10.3390/ijms21155506)
Supplement: Supplementary file 1 [file ijms-21-05506-s001.pdf]

Supplementary Materials

Table S1. Comparison of frequencies of genotypes in all analyzed mastocytosis patients (n = 234) and control (n = 5606).

	CHR	SNP	Gene	Mi	Ma	Genotypes				Recessive Inheritance Model				Dominant Inheritance Model			
				A1	A2	AFF	UNAFF	CHISQ	P	AFF	UNAFF	CHISQ	P	AFF	UNAFF	CHISQ	P
1	11	rs10838094	**OR51Q1**	A	G	4/51/179	932/2644/1914	171.5	5.71E-38	4/230	932/4558	38.24	6.24E-10	55/179	3576/1914	167.7	2.34E-38
2	9	rs80138802	**ABCA2**	C	A	8/33/155	2/267/5310	237.1	3.26E-52	8/188	2/5577	179.3	6.91E-41	41/155	269/5310	96.58	8.57E-23
3	14	rs11845537	**OTX2-AS1**	A	G	2/32/199	1/146/4899	108.3	3.00E-24	2/231	1/5045	27.57	1.51E-07	34/199	147/4899	91.75	9.85E-22
4	3	rs9828758		T	C	0/22/170	450/2301/2712	113.3	2.46E-25	0/192	450/5013	17.18	3.40E-05	22/170	2751/2712	112.3	3.07E-26
5	19	rs2279343	**CYP2B6**	G	A	11/15/193	356/2054/3162	89.56	3.56E-20	11/208	356/5216	0.6627	0.4156	26/193	2410/3162	85.14	2.78E-20
6	6	rs1611207	**HLA-V**	A	G	25/170/0	819/2618/2153	139.2	5.99E-31	25/170	819/4771	0.5068	0.4765	195/0	3437/2153	119.6	7.64E-28
7	1	rs76015112	**RPTN**	G	A	9/13/185	233/1767/3207	70.84	4.14E-16	9/198	233/4974	0.00751	0.9309	22/185	2000/3207	65.67	5.33E-16
8	1	rs1778155	**PDE4DIP**	T	C	50/145/0	1145/2562/1637	88.15	7.23E-20	50/145	1145/4199	1.976	0.1599	195/0	3707/1637	84.79	3.31E-20
9	21	rs61735841	**FTCD**	A	G	0/1/211	17/833/4302	39.18	3.10E-09	0/212	17/5135	0.7018	0.4022	1/211	850/4302	39.18	3.86E-10
10	19	rs34034473	ZNF134	C	A	0/52/181	4/514/4694	37.28	8.02E-09	0/233	4/5208	0.1789	0.6723	52/181	518/4694	36.47	1.55E-09
11	3	rs496250	FXR1	T	C	4/86/117	92/1294/4201	37.76	6.31E-09	4/203	92/5495	0.09998	0.7519	90/117	1386/4201	36.65	1.41E-09
12	6	rs3131382	MSH5	A	G	8/21/205	5/348/5251	115.4	8.58E-26	8/226	5/5599	112.1	3.44E-26	29/205	353/5251	13.64	0.000221
13	9	rs4316218		C	T	50/94/90	493/2352/2760	43.7	3.25E-10	50/184	493/5112	42.09	8.72E-11	144/90	2845/2760	10.45	0.001228
14	17	rs8081489		C	A	1/21/212	0/169/5099	44.98	1.71E-10	1/233	0/5268	22.52	2.08E-06	22/212	169/5099	25.65	4.10E-07
15	6	rs45487695	MOCS1	T	C	2/12/220	0/99/5395	60.17	8.59E-14	2/232	0/5494	46.97	7.20E-12	14/220	99/5395	20.29	6.66E-06
16	22	rs138366	CSNK1E	A	G	8/150/64	1407/2784/1403	55.35	9.56E-13	8/214	1407/4187	53.85	2.16E-13	158/64	4191/1403	1.591	0.2072
17	2	rs13015643	AC007682.1	C	A	5/56/172	40/766/4796	26.65	1.64E-06	5/228	40/5562	5.993	0.01436	61/172	806/4796	24.59	7.09E-07
18	11	rs7931273	ARHGAP42	T	C	8/84/142	571/2485/2549	25.28	3.24E-06	8/226	571/5034	11.52	0.000689	92/142	3056/2549	20.9	4.83E-06

Table S2. Comparison of frequencies of genotypes in patients with systemic mastocytosis (n = 93) and control (n = 5606).

	Chr	SNP	Gene	Min	Ma	Genotypes					Recessive Inheritance Model					Dominant Inheritance Model				
				A1	A2	AFF	UNAFF	CHISQ	DF	P	AFF	UNAFF	CHISQ	DF	P	AFF	UNAFF	CHISQ	DF	P
1	11	**rs10838094**	**OR51Q1**	A	G	4/22/67	932/2644/1914	55.89	2	7.30E-13	4/89	932/4558	10.5	1	0.00118	26/67	3576/1914	55.22	1	1.08E-13
2	6	**rs2857596**		C	A	22/34/31	313/2034/3209	63.18	2	1.91E-14	22/65	313/5243	59.3	1	1.39E-14	56/31	2347/3209	17.15	1	3.45E-05
3	9	**rs498404**		G	T	19/17/56	101/1328/4176	156	2	1.33E-34	19/73	101/5504	156	1	8.57E-36	36/56	1429/4176	8.81	1	0.002996
4	12	rs1479010	**NAV3**	T	C	35/47/11	989/2746/1868	33.2	2	6.19E-08	35/58	989/4614	24.8	1	6.45E-07	82/11	3735/1868	19.15	1	1.21E-05
5	3	rs9828758		T	C	0/7/53	450/2301/2712	35.86	2	1.64E-08	0/60	450/5013	5.38	1	0.02036	7/53	2751/2712	35.54	1	2.50E-09
6	16	rs9937881	**intergenic variant nearest tryptase-α and δ genes**	T	C	15/40/38	269/1802/3531	34.04	2	4.06E-08	15/78	269/5333	24.8	1	6.45E-07	55/38	2071/3531	19.22	1	1.16E-05
7	9	rs80138802	**ABCA2**	C	A	2/11/68	2/267/5310	80.52	2	3.27E-18	2/79	2/5577	66.9	1	2.80E-16	13/68	269/5310	21.26	1	4.01E-06
8	6	rs45487695	MOCS1	T	C	2/5/86	0/99/5395	124.7	2	8.22E-28	2/91	0/5494	118	1	1.57E-27	7/86	99/5395	16.1	1	6.00E-05
9	4	rs1820510	C4orf45	A	G	3/12/78	2/317/5287	115.5	2	8.30E-26	3/90	2/5604	106	1	6.63E-25	15/78	319/5287	18.07	1	2.13E-05
10	8	rs954009	FUC10	C	T	10/56/27	295/2072/3238	31.57	2	1.39E-07	10/83	295/5310	5.44	1	0.01966	66/27	2367/3238	30.88	1	2.75E-08
11	6	rs17404123	EYS	C	T	14/14/63	63/1067/4470	136.5	2	2.33E-30	14/77	63/5537	136	1	1.62E-31	28/63	1130/4470	6.197	1	0.0128
12	6	exm529784	CDSN	T	C	26/26/41	358/2066/3162	67.38	2	2.33E-15	26/67	358/5228	67.4	1	2.25E-16	52/41	2424/3162	5.831	1	0.01575

13	9	rs2230808	ABCA1	A	G	18/40/33	379/1985/3191	30.1	2	2.91E-07	18/73	379/5176	23	1	1.62E-06	58/33	2364/3191	16.4	1	5.14E-05
14	8	rs12546032	FUC10	A	G	10/55/28	288/2082/3235	29.49	2	3.95E-07	10/83	288/5317	5.82	1	0.01586	65/28	2370/3235	28.49	1	9.40E-08
15	4	rs2276938	FNIP2	G	C	3/10/79	2/287/5317	113.8	2	1.94E-25	3/89	2/5604	107	1	3.66E-25	13/79	289/5317	14.53	1	0.0001382
16	6	rs807816		G	A	12/13/68	41/923/4589	145.6	2	2.43E-32	12/81	41/5512	146	1	1.62E-33	25/68	964/4589	5.74	1	0.01659
17	1	rs12402123	CACNA1E	T	C	2/22/69	12/589/5005	31.06	2	1.80E-07	2/91	12/5594	14	1	0.00018	24/69	601/5005	21.32	1	3.88E-06
18	6	rs16895517	EYS	C	G	13/14/65	63/1037/4506	116.4	2	5.30E-26	13/79	63/5543	116	1	3.97E-27	27/65	1100/4506	5.396	1	0.02018
19	1	rs35701577	MEGF6	C	T	15/27/49	161/1613/3816	56.24	2	6.13E-13	15/76	161/5429	55.2	1	1.09E-13	42/49	1774/3816	8.56	1	0.003437
20	6	rs3131382	MSH5	A	G	7/4/82	5/348/5251	241.1	2	4.44E-53	7/86	5/5599	241	1	2.68E-54	11/82	353/5251	4.676	1	0.0306

Table S3. Comparison of frequencies of genotypes in all patients with all cutaneous mastocytosis (*n* = 141) and control (*n* = 5606).

	CHR	SNP	Gene	Mi	Ma	Genotypes					Recessive Inheritance Model					Dominant Inheritance Model				
				A1	A2	AFF	UNAFF	CHISQ	DF	P	AFF	UNAFF	CHISQ	DF	P	AFF	UNAFF	CHISQ	DF	P
1	9	rs80138802	**ABCA2**	C	A	6/22/87	2/267/5310	265.5	2	2.19E-58	6/109	2/5577	215.6	1	8.15E-49	28/87	269/5310	86.9	1	1.14E-20
2	11	rs10838094	**OR51Q1**	A	G	0/29/112	932/2644/1914	121.9	2	3.42E-27	0/141	932/4558	28.68	1	8.52E-08	29/112	3576/1914	118.6	1	1.31E-27
3	14	rs11845537	**OTX2-AS1**	A	G	1/23/116	1/146/4899	96.47	2	1.13E-21	1/139	1/5045	17.04	1	3.66E-05	24/116	147/4899	86.5	1	1.40E-20
4	3	rs9828758		T	C	0/15/117	450/2301/2712	79.08	2	6.72E-18	0/132	450/5013	11.82	1	0.0005847	15/117	2751/2712	78.4	1	8.43E-19
5	19	rs2279343	**CYP2B6**	G	A	6/3/124	356/2054/3162	73.89	2	9.01E-17	6/127	356/5216	0.7708	1	0.38	9/124	2410/3162	70.8	1	3.95E-17
6	1	rs76015112	**RPTN**	G	A	3/3/114	233/1767/3207	56.92	2	4.37E-13	3/117	233/4974	1.08	1	0.2986	6/114	2000/3207	55.77	1	8.15E-14
7	6	rs1611207	**HLA-V**	A	G	14/119/0	819/2618/2153	102.1	2	6.64E-23	14/119	819/4771	1.777	1	0.1825	133/0	3437/2153	82.12	1	1.28E-19
8	1	rs1778155	**PDE4DIP**	T	C	32/101/0	1145/2562/1637	62.08	2	3.30E-14	32/101	1145/4199	0.5337	1	0.465	133/0	3707/1637	58.11	1	2.48E-14
9	6	rs13195402	BTN2A1	T	G	2/23/116	0/382/4864	90.93	2	1.80E-20	2/139	0/5246	74.44	1	6.25E-18	25/116	382/4864	21.46	1	3.61E-06
10	1	rs973253	EGLN1	C	T	30/63/48	539/2277/2790	26.34	2	1.91E-06	30/111	539/5067	20.97	1	4.67E-06	93/48	2816/2790	13.61	1	0.0002253

Table S4. Comparison of frequencies of genotypes in adults' patients with cutaneous mastocytosis (n = 63) and control (n = 5606).

	CHR	SNP	Gene	Min	Ma	Genotypes					Recessive Inheritance Model					Dominant Inheritance Model				
				A1	A2	AFF	UNAFF	CHISQ	DF	P	AFF	UNAFF	CHISQ	DF	P	AFF	UNAFF	CHISQ	DF	P
1	9	rs80138802	**ABCA2**	C	A	4/7/40	2/267/5310	298.8	2	1.28E-65	4/47	2/5577	289.4	1	6.85E-65	11/40	269/5310	29.99	1	4.34E-08
2	14	rs11845537	**OTX2-AS1**	A	G	0/12/51	1/146/4899	54.19	2	1.71E-12	0/63	1/5045	0.01249	1	0.911	12/51	147/4899	53.72	1	2.31E-13
3	11	rs10838094	**OR51Q1**	A	G	0/18/45	932/2644/1914	39.29	2	2.95E-09	0/63	932/4558	12.85	1	0.0003371	18/45	3576/1914	36.47	1	1.55E-09
4	7	rs41271217	WDR60	G	A	0/12/51	5/247/5354	30.67	2	2.19E-07	0/63	5/5601	0.05624	1	0.8125	12/51	252/5354	29.71	1	5.01E-08

Table S5. Comparison of frequencies of genotypes in children with cutaneous mastocytosis (n = 78) and control (n = 5606).

	CHR	SNP	Gene	Mi	Ma	Genotypes					Recessive Inheritance Model					Dominant Inheritance Model				
				A1	A2	AFF	UNAFF	CHISQ	DF	P	AFF	UNAFF	CHISQ	DF	P	AFF	UNAFF	CHISQ	DF	P
1	9	rs80138802	ABCA2	C	A	2/15/47	2/267/5310	132.4	2	1.79E-29	2/62	2/5577	85.24	1	2.64E-20	17/47	269/5310	62.16	1	3.17E-15
2	11	rs10838094	OR51Q1	A	G	0/11/67	932/2644/1914	88.25	2	6.87E-20	0/78	932/4558	15.9	1	6.67E-05	11/67	3576/1914	87.39	1	8.90E-21
3	3	rs9828758		T	C	0/3/75	450/2301/2712	66.59	2	3.47E-15	0/78	450/5013	6.993	1	0.00818	3/75	2751/2712	66.55	1	3.42E-16
4	14	rs11845537	OTX2-AS1	A	G	1/11/65	1/146/4899	65.15	2	7.13E-15	1/76	1/5045	31.79	1	1.72E-08	12/65	147/4899	40.49	1	1.97E-10
5	1	rs76015112	RPTN	G	A	0/0/64	233/1767/3207	39.61	2	2.50E-09	0/64	233/4974	2.996	1	0.08346	0/64	2000/3207	39.61	1	3.10E-10
6	3	rs3218642	POLQ	T	G	2/12/64	7/299/5299	44.37	2	2.32E-10	2/76	7/5598	28.95	1	7.44E-08	14/64	306/5299	22.58	1	2.01E-06
7	18	rs8088340	RP11-945C19.1	C	T	1/10/67	2/192/5412	42.66	2	5.46E-10	1/77	2/5604	22.65	1	1.94E-06	11/67	194/5412	25.06	1	5.56E-07
8	8	rs1909936		C	T	1/14/63	4/312/5289	34.89	2	2.66E-08	1/77	4/5601	12.83	1	0.00034	15/63	316/5289	25.91	1	3.57E-07
9	19	rs2279343	CYP2B6	G	A	4/0/69	356/2054/3162	44.97	2	1.72E-10	4/69	356/5216	0.09986	1	0.752	4/69	2410/3162	42	1	9.12E-11
10	3	rs2728007		C	T	1/13/64	6/295/5270	28.09	2	7.96E-07	1/77	6/5565	8.572	1	0.00341	14/64	301/5270	22.99	1	1.62E-06
11	13	rs9530313	LINC00381	A	G	2/18/58	14/545/5035	30.48	2	2.41E-07	2/76	14/5580	14.64	1	0.00013	20/58	559/5035	20.55	1	5.81E-06

Tables legend: CHR—Chromosome, SNP—rs identifier of tested SNP, Gene—name of the gene in which tested SNP is located, A1 (Mi)—minor allele, A2 (Ma)—major allele, AFF—Genotypes/alleles in cases, UNAFF—Genotypes/alleles in controls, CHISQ—Chi-squatted statistic, DF—Degrees of freedom for test, P—Asymptotic p-value; GENOTYPES—provides a general test of association in the 2-by-3 contingency table of disease-by-genotype, RECESSIVE and DOMINANT INHERITANCE MODEL—provide a statistics for the minor allele in 2 by 2 contingency table (1df) in which A1A1 versus (A1A2, A2A2) and (A1A1, A1A2) versus A2A2, respectively, are tested.

Table S6. List of selected genes of cytokines, growth factors and transcriptions factors described as the potential factors in mastocytosis pathogenesis (3,4,6,8,9,11,14,15).

	**Genes**
Cytokines, growth factors and receptors	*IL-2, IL-2R, IL4, IL-4R, IL-6, IL6R, IL-9, IL9R, IL-10, IL-10R, IL-12,IL-12R, IL-13, IL-13R, IL-17, IL-22, IL-22R, IL-23, IL23R, IL-33, TNF, TNF-R, TGFB1, IFNG, SCF*
Cell signal transmission	*STAT3, STAT5A, STAT5B, STAT6, IL6ST*
Toll Like receptors	*TLR1, TLR2, TLR4, TLR5, TLR6, TLR10 and TLR11*
Transcription factors	*NFKB, TBX21, PRDM1, RUNX1, IRF-4, PPARG*
Apoptosis	*PDCD1, PDCD2, PGLG1, PGLG2,*
Treg genes	*FOXP3, CTLA-4, GITR*
MC-lymphocyte interaction proteins	*CD80, CD86, CD28, OX-40, OX-40L, CD40, CD40L, LGALS1*
Enzymes	*Neuropilin, IDO1, tryptase, chymase, apyrase, histidine decarboksylase, ENTPD1*
Histamine receptors	*HRH1, HRH2, HRH3, HRH4*
Oncogenes	*c-KIT*

Table S7. The list of genes involved in epigenetic processes, genes transcription and cell proliferation described as the potential factors involved in mastocytosis pathogenesis (18–21).

Process	Gene /Function	Frequency of Mutation *n* Advanced MC
Epigenetic process regulation	***TET-2*** - ***DNA*** demethylation	20-50%
	DNMT3A—DNA methyltransferase, CpG methylation	0-12%
	ASXL1—protein silencing chromatin and remodeling	14-25%
	IDH2—regulate histone methylation	0-4%
	HDAC—histone deacetylase	?
Cellular signal transmission	*JAK2,*	10-16%
	NRAS, KRAS	4-14%
	CBL	4-20%
Transcription factors	RUNX1<br>*ETV6*	10 -23%
RNA splicing factors	SRSF2,	43%
	SF3B1,U2AF, SRF3	0-9%
Oncogenes	TP53,	
	c-KIT	>90%

Table S8. Comparison of the frequency of polymorphisms of selected genes listed in tables S6 and S7 in examined group of mastocytosis patients and control.

CHR	SNP	A1	F_A	F_U	A2	CHISQ	P	OR	L95	U95	Overlapped gene	Annotation
14	rs5247	C	0,03205	0,01409	T	9,93	0,001626*	2,317	1,353	3,967	CMA1	coding nonsyn, intronic
2	rs16865650	T	0,1859	0,1361	C	9,378	0,002197	1,45	1,142	1,842	SF3B1	intronic
2	rs2227982	A	0	0,01944	G	9,273	0,002326	0	0	nan	PDCD1	coding nonsyn,3utr, coding *nonsyn
21	rs2834730	G	0,1346	0,1832	A	7,138	0,007545	0,6937	0,5297	0,9084	RUNX1	intronic
17	rs1625895	A	0,08333	0,1247	G	7,121	0,00762	0,6382	0,4576	0,89	TP53	intronic, non-coding intronic,5upstream,non-coding,3downstream
5	rs10471420	G	0,3184	0,2654	A	6,423	0,01126	1,293	1,059	1,577	IL6ST	intronic,5upstream, non-coding intronic
11	rs2229114	T	0,06897	0,04485	C	5,895	0,01519	1,577	1,088	2,286	IL10RA	coding nonsyn, non-coding,3utr,3downstream
16	rs1110470	C	0,5449	0,4902	T	5,375	0,02043	1,245	1,034	1,499	IL4R	intronic, non-coding intronic
21	rs2834709	T	0,4402	0,3878	C	5,172	0,02295	1,241	1,03	1,495	RUNX1	intronic
16	rs1805010	G	0,4765	0,4235	A	5,17	0,02298	1,239	1,03	1,491	IL4R	coding *nonsyn|,3utr, non-coding,5upstream
21	rs2268288	C	0,1674	0,2099	T	4,9	0,02686	0,7568	0,5909	0,9693	RUNX1	intronic,5upstream
21	rs2839634	C	0,4402	0,4923	T	4,888	0,02704	0,8108	0,673	0,9768	U2AF1	non-coding, intronic,
12	rs916041	A	0,5085	0,458	G	4,612	0,03174	1,224	1,018	1,473	ETV6	intronic, non-coding intronic
5	rs1295686	A	0,2927	0,2494	G	4,491	0,03407	1,246	1,016	1,527	IL-13	non-coding intronic, intronic
4	rs1020759	T	0,3568	0,4056	C	4,444	0,03502	0,813	0,6704	0,9859	NFKB1	intronic
3	rs1666988	A	0,2628	0,3076	G	4,244	0,03939	0,8025	0,6507	0,9897	HRH1	intronic
2	rs7575625	G	0,4295	0,3831	A	4,088	0,04319	1,212	1,006	1,462	DNMT3A	intronic
12	rs974728	G	0,4043	0,3584	A	4,045	0,04431	1,215	1,005	1,47	ETV6	intronic, non-coding intronic
6	rs8770	A	0,5427	0,4954	G	4,033	0,04461	1,209	1,004	1,456	PDCD2	3utr, non-coding,3downstream
16	rs113856625	A	0,1211	0,09294	G	3,981	0,04602	1,344	1,004	1,8	TPSG1	coding nonsyn, non-coding
2	rs7583409	G	0,3333	0,2906	A	3,959	0,04661	1,22	1,003	1,485	DNMT3A	intronic
12	rs729697	G	0,3846	0,3402	A	3,942	0,04709	1,212	1,002	1,466	ETV6	intronic, non-coding intronic
16	rs3024585	A	0,4893	0,4433	G	3,856	0,04958	1,203	1	1,448	IL4R	intronic,3downstream

*all listed in the table genes showed significant difference in the prevalence when analyzed separately. However, the analysis using the correction for the false discovery rate showed insignificant results.

Table S9. Comparison of the frequency of polymorphisms of selected genes listed in tables S6 and S7 in patients with systemic mastocytosis (ISM and SSM) and control.

CHR	SNP	A1	F_A	F_U	A2	CHISQ	P	OR	L95	U95	Overlapped Gene	Annotation
14	rs5247	C	0,04301	0,01409	T	10,66	**0,001094***	3,144	1,522	6,496	CMA1	coding nonsyn, intronic, PD
1	rs11465804	G	0,09239	0,04292	T	10,6	**0,001131**	2,27	1,367	3,77	IL23R	intronic, non-coding intronic
21	rs4817699	T	0,172	0,1021	C	9,685	**0,001858**	1,828	1,243	2,689	RUNX1	intronic
2	rs16865650	T	0,2151	0,1361	C	9,637	**0,001907**	1,74	1,221	2,479	SF3B1	intronic
16	rs113856625	A	0,1609	0,09294	G	9,292	**0,002301**	1,872	1,243	2,819	TPSG1	coding nonsyn, non-coding, PD
10	rs11188484	A	0,4301	0,3268	G	8,859	**0,002916**	1,555	1,16	2,084	ENTPD1	intronic, non-coding intronic
7	rs12699994	T	0,4462	0,3494	C	7,531	**0,006063**	1,5	1,121	2,009	HDAC9	intronic, non-coding intronic
10	rs4749926	A	0,2957	0,3939	G	7,405	**0,006504**	0,6459	0,4704	0,8871	IL2RA	intronic
21	rs2268288	C	0,1304	0,2099	T	6,92	**0,008523**	0,5647	0,3668	0,8694	RUNX1	intronic,5upstream
16	rs1110470	C	0,586	0,4902	T	6,723	**0,009516**	1,472	1,097	1,976	**IL4R**	intronic, non-coding intronic
7	rs2073963	G	0,4785	0,3872	T	6,422	**0,01127**	1,452	1,086	1,941	HDAC9	intronic, non-coding intronic
1	rs11209026	A	0,08602	0,04682	G	6,218	**0,01265**	1,916	1,139	3,222	IL23R	coding nonsyn,

21	rs2834730	G	0,1129	0,1832	A	6,068	**0,01377**	0,5676	0,3595	0,896	RUNX1	intronic
6	rs6923419	A	0,1613	0,1054	G	6,009	**0,01423**	1,632	1,099	2,423	PRDM1	intronic, non-coding intronic
3	rs6804441	G	0,09677	0,1625	A	5,839	**0,01567**	0,5522	0,3387	0,9001	CD80	intronic
12	rs974728	G	0,4451	0,3584	A	5,837	**0,0157**	1,436	1,069	1,928	ETV6	intronic, non-coding intronic
4	rs6839705	A	0,4892	0,4019	C	5,802	**0,01601**	1,426	1,067	1,905	TET2	intronic
5	rs10471420	G	0,3441	0,2654	A	5,786	**0,01615**	1,452	1,07	1,97	IL6ST	intronic,
10	rs4582902	T	0,5604	0,4726	C	5,543	**0,01855**	1,423	1,059	1,911	ENTPD1	intronic, non-coding intronic
12	rs743613	A	0,129	0,1977	G	5,46	**0,01946**	0,6013	0,3907	0,9254	ETV6	intronic
22	rs228941	C	0,3817	0,3026	G	5,411	**0,02001**	1,423	1,056	1,918	IL2RB	3utr,3downstream
7	rs10230371	C	0,5215	0,4374	T	5,257	**0,02186**	1,402	1,049	1,874	HDAC9	intronic, non-coding intronic
16	rs1004041	T	0,5217	0,4393	G	4,993	**0,02545**	1,392	1,04	1,864	TPSG1	coding nonsyn,3downstream
9	rs17804441	C	0,2688	0,2024	T	4,978	**0,02567**	1,449	1,044	2,01	CD274	intronic, non-coding intronic
2	rs13428812	G	0,3656	0,2908	A	4,956	**0,026**	1,406	1,04	1,9	DNMT3A	intronic
10	rs2051052	C	0,3763	0,4579	T	4,904	**0,02679**	0,7144	0,5298	0,9634	ENTPD1	3utr
1	rs10794642	T	0,4516	0,374	C	4,704	**0,03009**	1,379	1,03	1,845	IL22RA1	intronic
1	rs3795299	C	0,462	0,3835	G	4,702	**0,03013**	1,38	1,03	1,849	IL22RA1	coding nonsyn
9	rs1411262	A	0,2258	0,2991	G	4,7	**0,03016**	0,6835	0,4835	0,9661	CD274	intronic, non-coding intronic
16	rs3024585	A	0,5215	0,4433	G	4,535	**0,03321**	1,369	1,024	1,83	**IL4R**	intronic,3downstream
7	rs17349860	T	0,4032	0,3306	C	4,35	**0,03702**	1,368	1,018	1,838	HDAC9	non-coding intronic, intronic
2	rs7583409	G	0,3602	0,2906	A	4,287	**0,03841**	1,374	1,016	1,859	DNMT3A	intronic
7	rs10252945	A	0,3065	0,2409	C	4,28	**0,03857**	1,392	1,016	1,907	HDAC9	intronic, non-coding intronic
9	rs10481593	A	0,1667	0,231	G	4,271	**0,03877**	0,666	0,4518	0,9818	CD274	intronic, non-coding intronic
12	rs7311993	A	0,2527	0,1929	G	4,182	**0,04086**	1,415	1,013	1,976	ETV6	intronic, non-coding intronic
5	rs1859430	T	0,1774	0,2416	C	4,127	**0,0422**	0,677	0,4636	0,9886	IL9	intronic
17	rs1625895	A	0,07527	0,1247	G	4,117	**0,04245**	0,5714	0,3305	0,988	TP53	intronic, non-coding
12	rs3782137	T	0,4355	0,3645	C	3,972	**0,04626**	1,345	1,004	1,802	ETV6	intronic, non-coding intronic
4	rs1020759	T	0,3333	0,4056	C	3,971	**0,04629**	0,7326	0,5389	0,9961	NFKB1	intronic
16	rs3024613	T	0,4194	0,4923	C	3,898	**0,04833**	0,7447	0,5553	0,9988	**IL4R**	intronic,5upstream

*all listed in the table genes showed significant difference in the prevalence when analyzed separately. However, the analysis using the correction for the false discovery rate showed insignificant results.

Table S10. Comparison of the frequency of polymorphisms of selected genes listed in tables S6 and S7 in cutaneous patients (both children and adults) and controls.

CHR	SNP	A1	F_A	F_U	A2	CHISQ	P	OR	L95	U95	Overlapped Gene	Annotation
11	rs2229114	T	0,08511	0,04485	C	10,15	0,001444*	1,981	1,29	3,041	IL10RA	coding nonsyn, non-coding,3utr,3downstream
7	rs2243880	T	0,2624	0,2016	C	6,276	0,01224	1,409	1,076	1,844	HDAC9	intronic, non-coding intronic
18	rs11665084	T	0,1312	0,08821	C	6,255	0,01238	1,561	1,098	2,219	HRH4	coding nonsyn, intronic
18	rs11662595	G	0,1312	0,08883	A	6,038	0,014	1,549	1,09	2,202	HRH4	coding nonsyn
6	rs8770	A	0,5674	0,4954	G	5,707	0,0169	1,336	1,053	1,696	PDCD2	3utr, non-coding,3downstream
2	rs2227982	A	0	0,01944	G	5,589	0,01807	0	0	nan	PDCD1	coding nonsyn,3utr, coding *nonsyn
21	rs2839634	C	0,422	0,4923	T	5,444	0,01963	0,7528	0,5927	0,9563	U2AF1	non-coding, intronic,non-coding intronic,3downstream
1	rs3790565	C	0,1028	0,1523	T	5,243	0,02204	0,6381	0,433	0,9403	IL12RB2	intronic
3	rs1151999	A	0,4113	0,4797	C	5,158	0,02314	0,7578	0,5961	0,9634	PPARG	intronic, non-coding intronic
4	rs11466657	G	0,03546	0,0174	A	5,119	0,02367	2,076	1,087	3,964	TLR10	coding nonsyn,3downstream
3	rs1666988	A	0,2482	0,3076	G	4,566	0,03262	0,7432	0,5656	0,9767	HRH1	intronic
7	rs212666	A	0,3688	0,3093	G	4,544	0,03303	1,305	1,021	1,667	HDAC9	non-coding intronic, intronic
21	rs2834709	T	0,4504	0,3878	C	4,522	0,03346	1,293	1,02	1,64	RUNX1	intronic
5	rs34417936	T	0,03929	0,02098	C	4,367	0,03664	1,908	1,03	3,535	IL6ST	coding nonsyn,3utr,5upstream, intronic
19	rs401502	G	0,3007	0,3614	C	4,288	0,03838	0,7601	0,5858	0,9862	IL12RB1	coding nonsyn,3downstream
5	rs3756721	A	0,2411	0,193	G	4,072	0,0436	1,329	1,007	1,752	MEGF10	3utr, non-coding intronic
12	rs916041	A	0,5177	0,458	G	3,946	0,04699	1,27	1,003	1,609	ETV6	intronic, non-coding intronic
3	rs346087	A	0,2482	0,303	G	3,922	0,04766	0,7594	0,5779	0,998	HRH1	intronic
5	rs17164935	A	0,227	0,181	G	3,905	0,04813	1,329	1,001	1,763	MEGF10	coding nonsyn,3downstream, non-coding
21	rs3788054	T	0,156	0,2038	C	3,886	0,0487	0,7222	0,5219	0,9995	U2AF1	non-coding intronic, non-coding,intronic
7	rs17169602	A	0	0,01356	G	3,874	0,04903	0	0	nan	HDAC9	intronic, non-coding intronic

**all listed in the table genes showed significant difference in the prevalence when analyzed separately. However, the analysis using the correction for the false discovery rate showed insignificant results.

Table S11. The list of genes where differences in expression was described in indolent systemic mastocytosis patients (16).

Chromosome	Start	End	*Gene*	Description
1	32372022	32403988	*PTP4A2*	PTP4A2 protein tyrosine phosphatase 4A2
1	225589204	225616557	*LBR*	LBR lamin B receptor
2	64858755	64978048	*SERTAD2*	SERTA domain containing 2
2	65454829	65498387	*ACTR2*	ACTR2 actin related protein 2
2	187454058	187545629	*ITGAV*	integrin subunit alpha V
2	238536224	238690290	*LRRFIP1*	LRRFIP1 LRR binding FLII interacting protein 1
3	46395235	46402413	*CCR2*	CCR2 C-C motif chemokine receptor 2
3	178735011	178790067	*ZMAT3*	ZMAT3 zinc finger matrin-type 3
3	196433144	196439131	*C3ORF34*	CEP19 centrosomal protein 19
5	96211644	96255420	*LRAP*	ERAP2 endoplasmic reticulum aminopeptidase 2
5	138609441	138667366	*MATR3*	MATR3 matrin 3

7	139025196	139031065	*FMC1*	HSPC268 formation of mitochondrial complex V assembly factor 1 homolog
7	151832010	152133090	*MLL3*	KMT2C lysine methyltransferase 2C
8	71021997	71316062	*NCOA2*	NCOA2 nuclear receptor coactivator 2
8	144989321	145050913	*PELC1*	Plectin 1 intermediate filament binding protein 500 kDa
9	20341663	20622514	*MLLT3*	MLLT3 super elongation complex subunit
9	27546543	27573864	*C9ORF72*	C9orf72-SMCR8 complex subunit
9	74966341	74980163	*ZFAND5*	ZFAND5 zinc finger AN1-type containing 5
10	33189246	33247293	*ITGB1*	integrin subunit beta 1
11	108093559	108239829	*ATM*	ATM serine/threonine kinase
11	128328656	128457453	*ETS1*	ETS proto-oncogene 1 transcription factor
14	64150934	64194756	*SGPP1*	SGPP1 sphingosine-1-phosphate phosphatase 1
14	104029299	104057236	*C14ORF153*	COA8 cytochrome c oxidase assembly factor 8
15	55495164	55582034	*RAB27A*	RAB27A member RAS oncogene family
15	78857862	78887611	*CHRNA5*	CHRNA5 cholinergic receptor nicotinic alpha 5 subunit
15	102501016	102516808	*FAM39DP*	WASH3P WASP family homolog 3 pseudogene
16	48394442	48419229	*SIAH1*	SIAH1 siah E3 ubiquitin protein ligase 1
17	79648224	79650954	*ARL16*	ADP ribosylation factor like GTPase 16
22	45064427	45133561	*PRR5*	PRR5 proline rich 5

Table S12. Comparison of the frequency of polymorphisms of selected genes listed in tables S11 in examined group of mastocytosis patients and control.

CHR	SNP	A1	F_A	F_U	A2	CHISQ	P	OR	L95	U95	Overlapped Gene	Annotation
9	rs10122902	A	0,1517	0,2127	G	10,05	0,00152*	0,6621	0,5123	0,8558	C9orf72	coding syn, non-coding
15	rs1014597	G	0,2768	0,3409	T	8,217	0,004149	0,7399	0,6018	0,9098	RAB27A	intronic
15	rs2229961	A	0	0,01508	G	7,16	0,007453	0	0	nan	CHRNA5	coding nonsyn,5upstream
9	rs12347222	T	0,1921	0,2522	G	7,071	0,007836	0,705	0,5442	0,9133	C9orf72	intronic, non-coding intronic,3utr,3downstream
15	rs2444039	T	0,312	0,3681	G	6,108	0,01346	0,7783	0,6376	0,9499	RAB27A	intronic, non-coding intronic
9	rs6475430	C	0,09615	0,06752	T	5,761	0,01639	1,469	1,071	2,015	MLLT3	intronic, non-coding intronic
22	rs5765930	A	0,1368	0,177	G	5,021	0,02504	0,7368	0,5635	0,9634	PRR5	intronic, non-coding intronic
15	rs549774	G	0,4103	0,4617	A	4,794	0,02856	0,8109	0,672	0,9786	RAB27A	intronic, non-coding intronic
9	rs2780840	G	0,1109	0,1483	A	4,747	0,02935	0,7163	0,5299	0,9683	MLLT3	intronic, non-coding intronic

*all listed in the table genes showed significant difference in the prevalence when analyzed separately. However, the analysis using the correction for the false discovery rate showed insignificant results.

Table S13. Comparison of the frequency of polymorphisms of selected genes listed in tables S11 in systemic mastocytosis (ISM and SSM) patients and control.

CHR	SNP	A1	F_A	F_U	A2	CHISQ	P	OR	L95	U95	Overlapped Gene	Annotation
8	rs35858667	A	0,09677	0,05102	G	7,805	0,00521	1,993	1,217	3,264	PLEC	coding nonsyn,3downstream
2	rs2029770	C	0,2473	0,181	T	5,396	0,02018	1,486	1,062	2,081	LRRFIP1	intronic, non-coding intronic
11	rs11221342	C	0,2258	0,1639	T	5,092	0,02404	1,488	1,051	2,106	ETS1	intronic, non-coding intronic
9	rs10122902	A	0,1452	0,2127	G	4,999	0,02537	0,6287	0,417	0,9477	C9orf72	coding syn, non-coding
7	rs10265	G	0,2826	0,2173	T	4,518	0,03354	1,419	1,026	1,962	C7orf55	non-coding intronic, coding nonsyn
2	rs12995190	C	0,1559	0,2201	A	4,401	0,03591	0,6547	0,4394	0,9753	LRRFIP1	intronic, non-coding intronic

*all listed in the table genes showed significant difference in the prevalence when analyzed separately. However, the analysis using the correction for the false discovery rate showed insignificant results.

Table S14. Comparison of the frequency of polymorphisms of selected genes listed in tables S11 in cutaneous (both children and adults) patients and control.

CHR	SNP	A1	F_A	F_U	A2	CHISQ	P	OR	L95	U95	Overlapped Gene	Annotation
15	rs1014597	G	0,2518	0,3409	T	9,766	0,001778*	0,6504	0,4957	0,8535	RAB27A	intronic
9	rs7041793	G	0,09712	0,05612	A	8,468	0,003615	1,809	1,207	2,713	MLLT3	intronic, non-coding intronic
9	rs6475430	C	0,1064	0,06752	T	6,515	0,0107	1,644	1,118	2,418	MLLT3	intronic, non-coding intronic
15	rs2444039	T	0,2979	0,3681	G	5,849	0,01559	0,7282	0,5625	0,9426	RAB27A	intronic, non-coding intronic
9	rs7870252	G	0,2695	0,211	A	5,631	0,01765	1,38	1,056	1,802	MLLT3	intronic, non-coding intronic
9	rs10122902	A	0,156	0,2127	G	5,296	0,02138	0,6844	0,4946	0,9471	C9orf72	coding syn, non-coding
2	rs4513276	T	0,3617	0,2995	G	5,064	0,02443	1,326	1,036	1,696	ACTR2	3utr
2	rs7589984	T	0,234	0,2955	C	5,012	0,02518	0,7283	0,5512	0,9624	ACTR2	intronic, non-coding intronic
9	rs2780840	G	0,1014	0,1483	A	4,703	0,03011	0,6486	0,4373	0,9621	MLLT3	intronic, non-coding intronic
22	rs5765930	A	0,1277	0,177	G	4,614	0,03171	0,6807	0,4782	0,9689	PRR5	intronic, non-coding intronic
11	rs1800057	G	0,02837	0,01338	C	4,557	0,03278	2,153	1,047	4,428	ATM	coding *nonsyn, coding nonsyn,3downstream
9	rs636100	G	0,1	0,1446	A	4,416	0,0356	0,6573	0,4432	0,9749	MLLT3	intronic, non-coding intronic
15	rs2229961	A	0	0,01508	G	4,316	0,03776	0	0	nan	CHRNA5	coding nonsyn,5upstream
9	rs12347222	T	0,1953	0,2522	G	4,308	0,03794	0,7196	0,5268	0,9832	C9orf72	intronic, non-coding intronic,3utr,3downstream
2	rs11424	G	0,3587	0,3019	C	4,113	0,04255	1,293	1,008	1,66	ACTR2	3utr
2	rs17703416	G	0,3617	0,3063	A	3,96	0,04659	1,283	1,003	1,641	ACTR2	intronic

*all listed in the table genes showed significant difference in the prevalence when analyzed separately. However, the analysis using the correction for the false discovery rate showed insignificant results.

Tables legend: CHR—chromosome, SNP—rs identifier of tested SNP, BP—base pair based on the human reference genome GRCh37, A1—minor allele, A2—major allele, F_A—frequency of A1 allele in patients, F_U—frequency of A1 allele in controls, CHISQ—Basic allelic test chi-square (1df), P—*p*-value for CHISQ, OR—odds ratio, SE—Standard Error, L95 and U95—95% confidence interval for odds ratio, lower bound and upper bound, respectively.

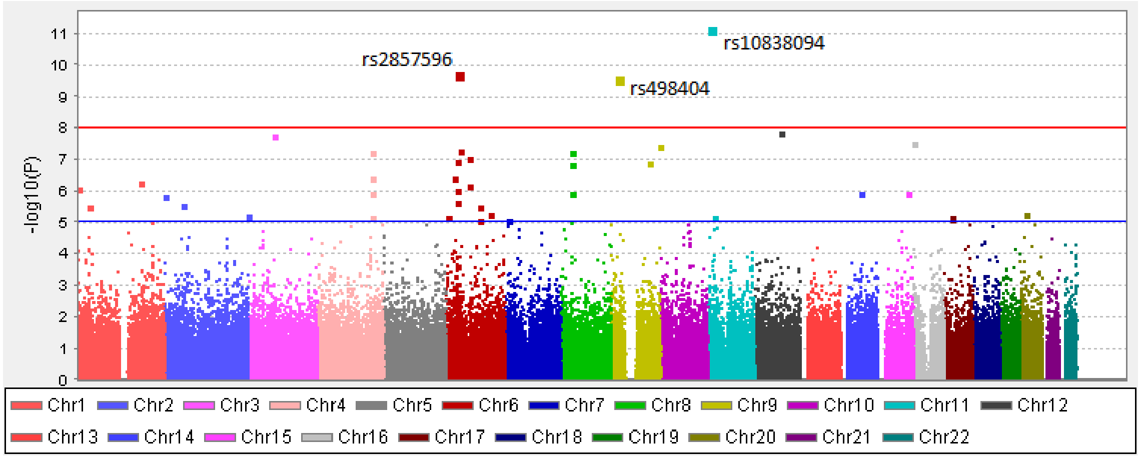

Figure S1. The 'Manhattan' plot for the genome-wide association study of systemic mastocytosis patients. On the x-axis, each color represents different chromosome. The $-\log_{10}$ of the unadjusted *p*-values (without multiple testing corrections) are shown on the y-axis. Blue line indicates the suggestive association threshold (10^{-5}), while red line indicates genome-wide significant threshold (10^{-8}).

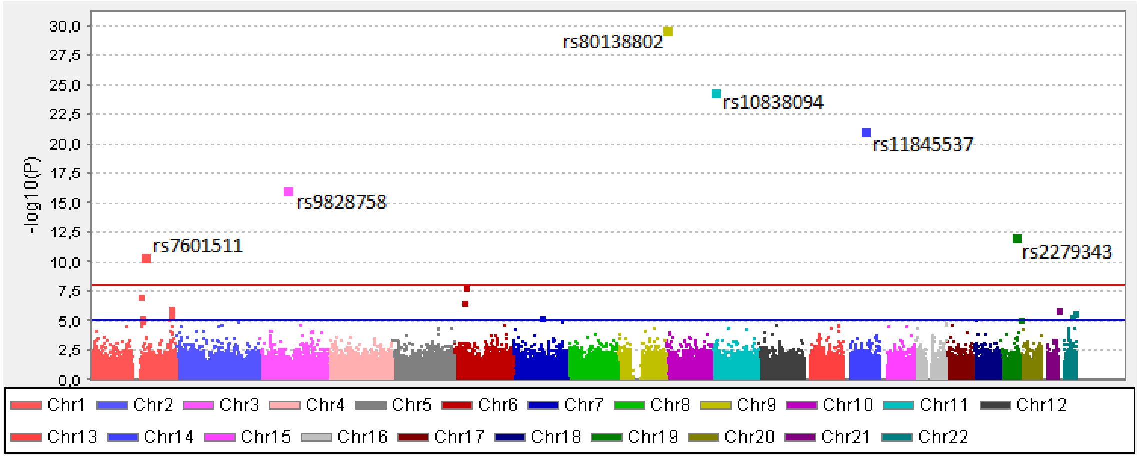

Figure S2. The 'Manhattan' plot for the genome-wide association study of cutaneous mastocytosis patients. On the x-axis, each color represents different chromosome. The $-\log_{10}$ of the unadjusted *p*-values (without multiple testing corrections) are shown on the y-axis. Blue line indicates the suggestive association threshold (10^{-5}), while red line indicates genome-wide significant threshold (10^{-8}).

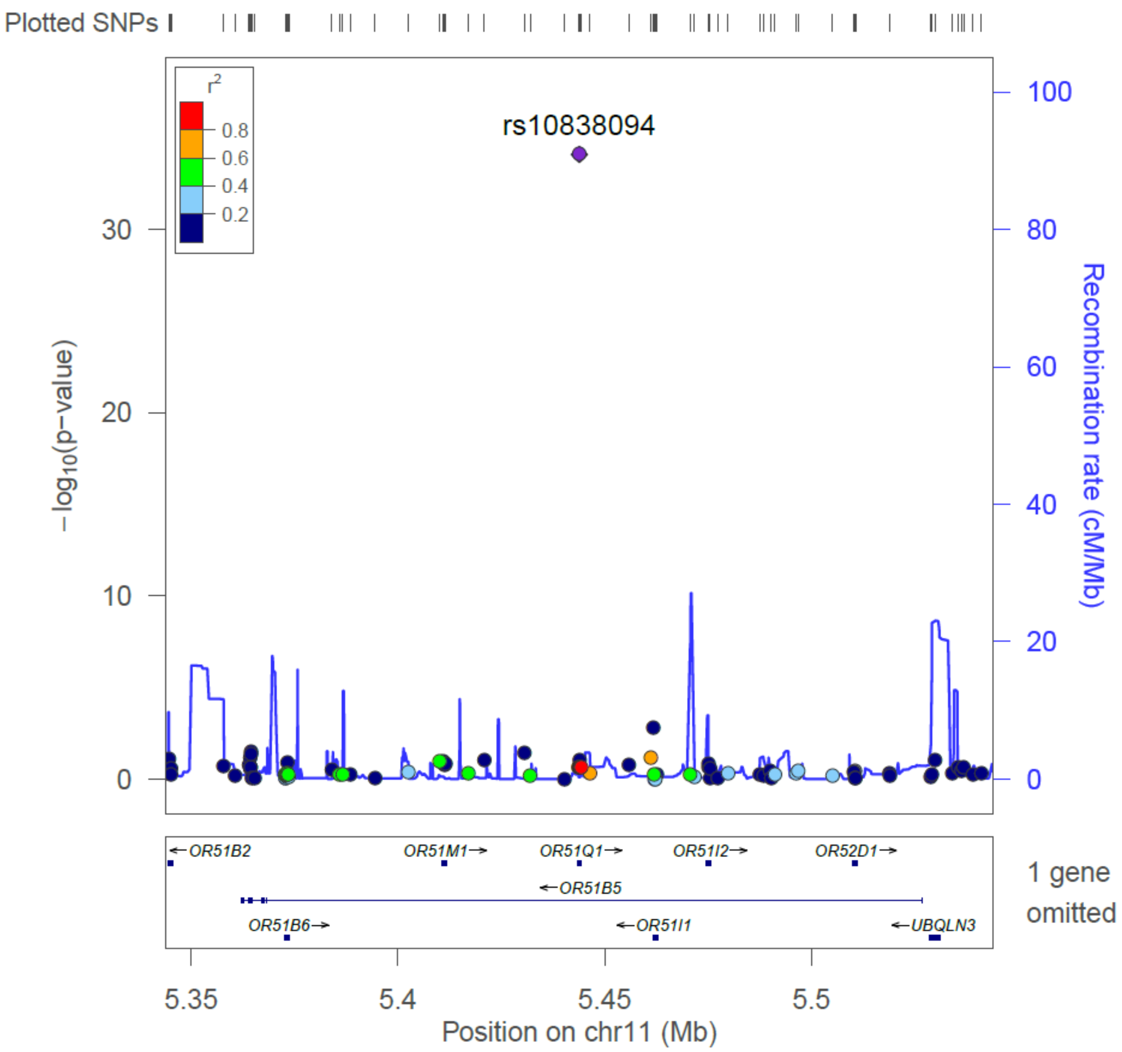

Figure S3. LocusZoom plot for rs10838094 located in *OR51Q1* gene. One of the major protective factors for developing mastocytosis OR = 0.2071 (95% CI; 0.1572–0.2728). $p = 2.21 \times 10^{-29}$.

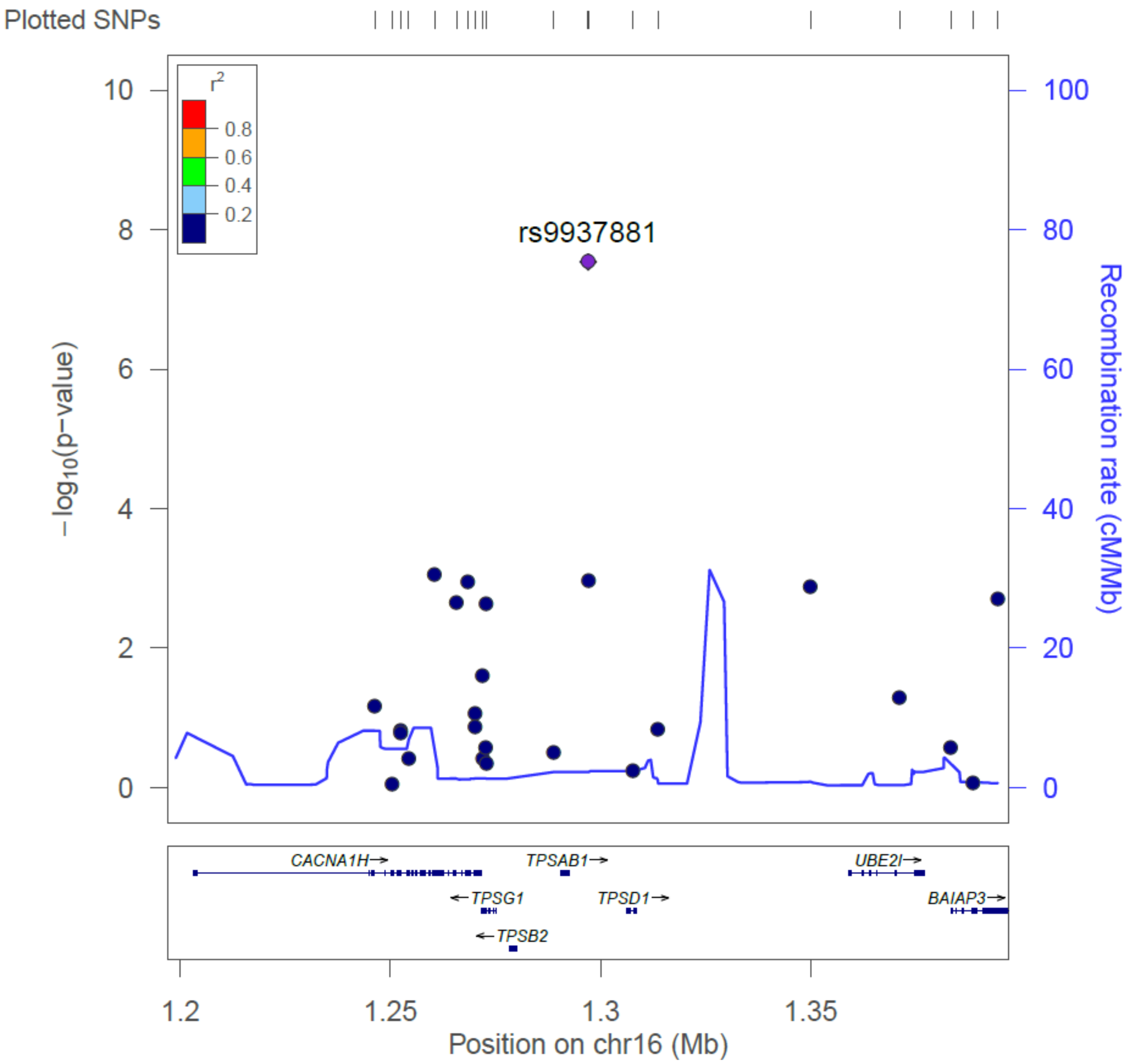

Figure S4. LocusZoom plot for rs9937881 in chromosome 16 located near *TPSABA1* and *TPSD*1 genes. The risk factor for developing systemic mastocytosis: OR = 2.697 (95% CI; 0.1634–3.716). $p = 7.38 \times 10^{-5}$.
